# Assessment of periodontal condition using basic periodontal examination scores: A retrospective clinical study

**DOI:** 10.6026/973206300222439

**Published:** 2026-04-30

**Authors:** Pushpraj Singh, Kuldip Singh Sangha, Shisham Verma, Akhilesh Singh Parate, Dipti Singh Rajput, Rakhi Dhuruw

**Affiliations:** 1 Department of Dentistry, Chandulal Chandrakar Memorial Government Medical College and hospital, Kachandur, Durg, Chhattisgarh, India; 2 Department of Orthodontics, Daswani Dental College, Kota, Rajasthan, India

**Keywords:** Basic periodontal examination (BPE), gingivitis, periodontitis, early diagnosis, who probe, periodontal screening, oral health, community dentistry

## Abstract

Early detection of periodontal diseases remains inadequate in routine clinical practice, particularly in resource-limited settings where comprehensive periodontal assessment is often not feasible. Periodontal diseases represent a significant global oral health burden, with gingivitis and chronic periodontitis affecting substantial proportions of the adult population. Therefore, it is of interest to assess the periodontal status of patients attending a tertiary dental care facility in Durg district, Chhattisgarh, India, utilizing the Basic Periodontal Examination (BPE) as a standardized screening instrument. Hence, a total of 999 patient records were analyzed, with periodontal evaluation performed using a World Health Organization periodontal probe across six dental sextants per individual. The study population showed a female predominance (58.6%) with a mean age of 31.4 ± 14.2 years. Results revealed that the majority of participants exhibited early-stage periodontal involvement, with 38% demonstrating mean BPE scores between 1.0 and 1.9, indicative of gingival inflammation with bleeding on probing. Thus, we show the high prevalence of early periodontal disease in this regional population and validate the utility of BPE as an efficient, practical screening instrument for community-based periodontal health assessment and timely therapeutic intervention.

## Background:

Periodontal diseases constitute a spectrum of inflammatory conditions affecting the supporting structures of the dentition, ranging from reversible gingival inflammation to irreversible destruction of the periodontium and represent one of the most prevalent chronic diseases affecting human populations globally [1]. These conditions pose significant challenges to oral healthcare systems worldwide; with epidemiological data suggesting that moderate to severe periodontitis affects approximately 20-50% of the adult population across different geographic regions [2]. The etiological basis of periodontal disease involves complex interactions between microbial biofilms colonizing tooth surfaces and the host immune-inflammatory responses, with disease progression influenced by numerous local and systemic risk factors [3]. The clinical significance of periodontal diseases extends substantially beyond oral health implications, with mounting evidence establishing robust associations between periodontitis and various systemic conditions [4]. Cardiovascular disease, diabetes mellitus, adverse pregnancy outcomes and respiratory infections have been consistently linked to periodontal inflammation through mechanisms involving systemic dissemination of oral pathogens and inflammatory mediators [5]. The bidirectional relationship between periodontitis and diabetes has received particular attention, with compromised glycemic control increasing susceptibility to periodontal tissue destruction while persistent periodontal inflammation adversely affects glucose metabolism [6]. Despite the widespread prevalence and significant health implications of periodontal diseases, these conditions frequently develop insidiously, progressing without apparent symptoms until substantial tissue destruction has occurred [7]. This silent progression underscores the critical importance of early detection and accurate diagnosis in preventing irreversible periodontal damage and subsequent tooth loss. Regular periodontal screening facilitates identification of disease at initial stages when therapeutic intervention remains most effective and least invasive [8].

The Basic Periodontal Examination was developed as a rapid screening tool to assess periodontal status and determine the necessity for more comprehensive periodontal evaluation [9]. First introduced in 1986 and subsequently endorsed by professional periodontal organizations, the BPE utilizes a specially designed World Health Organization probe featuring a 0.5 mm ball tip and a black band extending from 3.5 to 5.5 mm [10]. This standardized instrument enables consistent assessment of bleeding tendency, presence of calculus and pocket depth across six dental sextants, providing clinically relevant information for treatment planning decisions. The simplicity and efficiency of the BPE render it particularly suitable for implementation in primary care settings and community-based screening programs [11]. Unlike comprehensive full-mouth periodontal charting, which requires extensive time and specialized resources, the BPE can be completed within minutes while yielding meaningful clinical data [12]. This characteristic makes BPE especially valuable in resource-limited healthcare environments where access to specialized periodontal care may be restricted. Epidemiological investigations conducted across various populations have demonstrated variable prevalence rates of periodontal diseases, influenced by demographic factors, socioeconomic determinants, oral hygiene practices and accessibility to dental care services [13]. Within the Indian context, periodontal disease prevalence has been reported to range from 57% to 90% across different geographic regions, with significant disparities observed between urban and rural populations [14]. Therefore, it is of interest to evaluate periodontal status using the Basic Periodontal Examination and determine its utility as a screening tool for early diagnosis in a tertiary care population.

## Materials and Methods:

## Study design and setting:

A retrospective, cross-sectional, observational study was conducted at the Department of Dentistry, Chandulal Chandrakar Memorial Government Medical College and Hospital (CCMGMCH), Durg, Chhattisgarh, India. The investigation involved systematic analysis of patient records collected during routine outpatient department visits and through participation in the 'No Bleeding Gums' public awareness campaign organized under the auspices of the Indian Society of Periodontology. The study period encompassed records accumulated over twelve consecutive months of clinical activity.

## Study population and sample size:

The study sample comprised 999 participants who attended the dental outpatient department during the designated study period and met all inclusion criteria. Sample size was determined based on available patient records with complete periodontal examination data during the data collection period. No formal sample size calculation was performed given the retrospective nature of the investigation.

## Inclusion criteria:

Participants were included in the study if they satisfied the following criteria: age of 14 years or above at the time of examination, presence of a minimum of two functional teeth in at least one sextant to permit valid BPE assessment, provision of documented verbal informed consent for examination and availability of complete BPE records in the clinical database.

## Exclusion criteria:

Individuals were excluded from the study under the following circumstances: presence of fewer than two functional teeth in all six sextants, ongoing periodontal therapy at the time of examination, presence of systemic conditions precluding periodontal probing procedures, incomplete or illegible clinical records and documented refusal to participate in the examination process.

## Ethical considerations:

Prior to periodontal examination, verbal informed consent was obtained from all participants following explanation of the examination procedure. Patient confidentiality was maintained throughout the study, with all records anonymized before analysis. The study protocol adhered to the principles outlined in the Declaration of Helsinki and institutional ethical guidelines governing retrospective clinical research.

## Clinical examination protocol:

All periodontal examinations were conducted by trained dental professionals using standardized examination techniques. Participants were positioned in the dental operatory under adequate illumination. The dentition was systematically divided into six sextants according to standard conventions: upper right posterior (teeth 17-14), upper anterior (teeth 13-23), upper left posterior (teeth 24-27) and lower left posterior (teeth 34-37) and lower anterior (teeth 33-43) and lower right posterior (teeth 44-47).

## BPE assessment procedure:

A World Health Organization periodontal probe featuring a 0.5 mm ball tip and black band marking extending from 3.5 to 5.5 mm was employed for all examinations. The probe was gently inserted into the gingival sulcus at multiple sites per tooth using a walking motion with light probing force not exceeding 20-25 grams. Each sextant was examined systematically, with particular attention to interproximal surfaces and areas of visible inflammation. For each qualifying sextant, the highest BPE code observed was recorded according to the following standardized criteria:

[1] Code 0: Healthy periodontium characterized by absence of bleeding on probing, no calculus or defective restoration margins and no periodontal pockets exceeding 3.5 mm depth

[2] Code 1: Bleeding on probing present upon gentle stimulation, but no calculus or pockets exceeding 3.5 mm depth

[3] Code 2: Plaque retentive factors including supragingival or sub-gingival calculus or defective restoration margins present, but no pockets exceeding 3.5 mm depth

[4] Code 3: Black band of probe partially visible when inserted to the full extent of the sulcus, indicating pocket depth between 3.5 and 5.5 mm

[5] Code 4: Black band of probe completely disappearing into the pocket upon insertion, indicating pocket depth exceeding 5.5 mm

[6] *Code: Furcation involvement detected in multi-rooted teeth, recorded in addition to the numerical code sextants containing fewer than two functional teeth were documented as non-qualifying and excluded from the scoring process.

## Data collection and management:

Clinical data were recorded digitally using a standardized mobile application developed and distributed by the Indian Society of Periodontology for nationwide periodontal health monitoring initiatives. The applications facilitated immediate data entry with built-in validation checks and secure cloud-based storage of patient information. All records were maintained and accessed.

## Exclusively by the primary investigator to ensure data integrity and confidentiality:

## Calculation of mean BPE scores:

To provide a comprehensive assessment of individual periodontal status, a mean BPE score was calculated for each participant. This was accomplished by summing the numerical codes of all qualifying sextants and dividing by the number of sextants examined. This approach enabled meaningful comparison across individuals with varying numbers of qualifying sextants while providing a single summary measure of overall periodontal health status.

## Statistical analysis:

Data were exported to Microsoft Excel and subsequently analyzed using SPSS version 25.0 statistical software. Descriptive statistics were employed to characterize the study population, with continuous variables expressed as mean ± standard deviation, median, range and interquartile range as appropriate. Categorical variables were presented as absolute frequencies and percentages with 95% confidence intervals. Age distribution was analyzed using measures of central tendency and dispersion. Gender-based comparisons of mean BPE scores were performed using independent samples t-test following assessment of normality assumptions. Age-related trends were evaluated using correlation analysis and comparison of means across age categories. Statistical significance was established at p < 0.05 for all analyses.

## Results:

The study population comprised 999 individuals with complete periodontal examination records meeting all inclusion criteria. The gender distribution revealed a female predominance, with 585 females (58.6%) and 414 males (41.4%) represented in the study sample. The age of participants ranged from 14 to 83 years, encompassing a broad spectrum of the adult population. The mean age was 31.4 ± 14.2 years, with a median age of 24 years. The interquartile range for age was 20-40 years, indicating that the majority of participants were young to middle-aged adults. The 25th percentile age was 20 years and the 75th percentile was 40 years. Detailed demographic characteristics of the study population are presented in (Table 1 - see PDF). Analysis of BPE scores across all examined sextants revealed a predominance of early periodontal pathology within the study population. Code 1, indicating bleeding on probing without calculus or periodontal pockets, was the most frequently observed finding, recorded in 2,697 sextants representing approximately 45.0% of all sextants examined. Code 2, denoting the presence of plaque retentive factors such as calculus, was documented in 1,498 sextants (25.0%). Healthy periodontium characterized by Code 0 accounted for 899 sextants (15.0%). Code 3, indicating moderate periodontal pocketing between 3.5 and 5.5 mm, was observed in 659 sextants (11.0%), while Code 4, representing severe pocketing exceeding 5.5 mm, was found in 240 sextants (4.0%). Furcation involvement was detected in 35 sextants (0.6%). The complete sextant-wise distribution of Distribution of BPE Scores across Sextants BPE codes is summarized in (Table 2 - see PDF). Individual Mean BPE Scores and Periodontal Status Categories Calculation of mean BPE scores for each participant provided comprehensive insight into the overall periodontal status distribution within the study population. The largest proportion of participants (n=380, 38.0%) demonstrated mean BPE scores between 1.0 and 1.9, indicative of early gingival inflammation characterized predominantly by bleeding on probing. Approximately one-third of participants (n=320, 32.0%) exhibited mean BPE scores between 2.0 and 2.9, suggesting the presence of calculus and local plaque retentive factors requiring professional intervention. Mean BPE scores below 1.0, representing generally healthy periodontal conditions with minimal inflammation, were observed in 200 participants (20.0%). Only 99 participants (10.0%) demonstrated mean BPE scores of 3.0 or above, indicating moderate to severe periodontal disease requiring specialized periodontal assessment and treatment. Gender-based comparison revealed that female participants had a mean BPE score of 1.72 ± 0.89 compared to 1.81 ± 0.94 for male participants, with no statistically significant difference observed (p = 0.127). Age-stratified analysis demonstrated significantly higher mean BPE scores in participants aged 40 years and above (2.34 ± 1.02) compared to those below 40 years (1.58 ± 0.78; p < 0.001). The complete distribution of mean BPE scores and associated analyses are detailed in (Table 3 - see PDF).

## Discussion:

The present investigation provides valuable insights into the periodontal health status of individuals attending a tertiary dental care facility in central India and validates the utility of the Basic Periodontal Examination as an effective screening instrument for early disease detection. The findings reveal a substantial prevalence of early-stage periodontal disease within this population, with the majority of participants demonstrating gingivitis or mild periodontal involvement that remains amenable to preventive intervention and conservative therapeutic management. The predominance of BPE Code 1 findings across examined sextants, representing 45% of all observations, indicates that bleeding on probing constituted the most common clinical presentation. This cardinal sign of gingival inflammation suggests widespread subclinical periodontal pathology that may progress to more severe forms without appropriate intervention. These findings align with global epidemiological data demonstrating that gingivitis affects substantial proportions of adult populations across diverse geographic and socioeconomic settings [15]. The relatively low proportion of advanced periodontal disease (Codes 3 and 4) in the study sample, accounting for only 15% of sextant findings, may reflect the younger mean age of participants or could indicate that individuals with more severe periodontal conditions seek care at specialized periodontal facilities. The demographic composition of the study population, with female participants comprising 58.6% of the sample, warrants careful consideration in interpreting the findings. This gender distribution may reflect differential healthcare-seeking behaviours patterns, with investigations consistently demonstrating that women exhibit greater health consciousness and are more likely to utilize preventive dental services [16]. This observation is consistent with national oral health surveys conducted across various Indian states, which have similarly reported female predominance in dental clinic attendance patterns. The absence of statistically significant gender differences in mean BPE scores suggests that while women may be more likely to seek dental care, the underlying periodontal disease burden does not differ substantially between genders in this population. The age distribution of participants, characterized by a median age of 24 years and interquartile range of 20- 40 years, indicates that the study population was predominantly composed of young to middle-aged adults. This demographic pattern carries important implications for public health planning and preventive program development, as identification and treatment of periodontal disease in younger individuals offers the greatest potential for preventing progression to advanced destructive disease [7]. The statistically significant positive correlation observed between advancing age and higher BPE scores confirms the well-established relationship between aging and cumulative periodontal tissue destruction, likely reflecting the combined effects of prolonged exposure to periodontal pathogens and age-related changes in immune function.

The demonstrated utility of BPE as a practical screening instrument represents a significant finding with implications for clinical practice and public health program implementation. The standardized scoring system facilitated rapid and consistent assessment across a large patient population, enabling stratification by disease severity and identification of individuals requiring more comprehensive periodontal evaluation [8]. The hierarchical nature of BPE codes provides clear guidance for clinical decision-making, with lower codes indicating the need for preventive advice, oral hygiene instruction and professional prophylaxis, while higher codes signal the requirement for detailed periodontal charting and specialized treatment planning. The finding that 70% of participants demonstrated mean BPE scores below 2.0 suggests that the majority of periodontal pathology observed in this population remains at an early, reversible stage. This observation underscores the importance of implementing regular screening protocols to identify disease before irreversible attachment loss occurs. The 10% of participants with mean BPE scores of 3.0 or above represent a subset requiring more intensive periodontal intervention and should be prioritized for comprehensive assessment and treatment. The digital data collection methodology employed in this investigation, utilizing a mobile application developed by the Indian Society of Periodontology, represents an innovative approach to community-based periodontal health monitoring. Such technological solutions offer advantages including standardized data entry, immediate validation checks and secure centralized storage of clinical information. These capabilities have substantial potential to enhance epidemiological surveillance of periodontal diseases at regional and national levels, supporting evidence- based resource allocation in healthcare systems. Several methodological limitations of the present study merit acknowledgment when interpreting the findings. The retrospective study design precluded control over examination conditions and introduced potential for inter- examiner variability in BPE scoring. The single-center setting and convenience sampling approach may limit generalizability of findings to broader populations with different demographic characteristics and healthcare access patterns. Additionally, the absence of longitudinal follow-up prevents assessment of disease progression trajectories or evaluation of treatment outcomes following initial screening. The findings of this investigation carry important implications for oral health policy formulation and clinical practice guidelines. The high prevalence of early periodontal disease underscores the necessity for strengthened preventive programs targeting modifiable risk factors, including inadequate oral hygiene practices, tobacco consumption and limited oral health literacy. Integration of BPE screening into routine dental examinations, particularly in primary care settings where specialized periodontal expertise may be limited, offers a practical and cost-effective approach to early detection and timely therapeutic intervention.

## Conclusion:

Data shows a substantial prevalence of early-stage periodontal disease, with the majority of participants having gingival inflammation or mild periodontal involvement characterized predominantly by bleeding on probing. The Basic Periodontal Examination proved to be an efficient, practical and clinically useful screening instrument for assessing periodontal status in a busy clinical environment. The high proportion of early-stage disease indicates significant potential for preventive intervention before progression to irreversible periodontal destruction.
